# Co-Doped Nanoporous Fe_3_P Self-Supported Electrodes for Enhanced Alkaline Hydrogen Evolution

**DOI:** 10.3390/nano16120761

**Published:** 2026-06-17

**Authors:** Nana Yang, Ning Mi, Lin Lei, Kang Xi, Furong Xu, Haorui Liu

**Affiliations:** 1 School of Materials Engineering, Longdong University, Qingyang 745000, China; 2 School of Mechanical Engineering, Lanzhou Petrochemical University of Vocational Technology, Lanzhou 730060, China; 3 School of Materials Science and Engineering, Lanzhou University of Technology, Lanzhou 730050, China

**Keywords:** hydrogen evolution reaction, Fe-based phosphides, dealloying, Co doping

## Abstract

Transition-metal phosphides are promising non-noble-metal electrocatalysts for alkaline hydrogen evolution, yet further improving their performance remains challenging. In this work, a Co-doped nanoporous Fe_3_P self-supported electrode was fabricated by vacuum high-frequency induction and melt spinning of Fe_75_Co_5_P_20_ precursor alloys, followed by electrochemical dealloying. Nanoporous Fe_3_P prepared from Fe_80_P_20_ was used as the reference. Structural analyses show that dealloying selectively removes the α-Fe phase while preserving the Fe_3_P framework, resulting in a three-dimensional nanoporous architecture. XPS results further confirm successful Co incorporation and reveal that Co doping modifies the local chemical environment of Fe and P. Benefiting from the combined effects of Co incorporation and the nanoporous structure, np-Co-Fe_3_P exhibits significantly improved HER performance in 1.0 M KOH, requiring only 70 mV to reach 10 mA cm^−2^, much lower than that of np-Fe_3_P (199 mV). In addition, np-Co-Fe_3_P shows a smaller Tafel slope of 94 mV dec^−1^, lower charge-transfer resistance, and a larger double-layer capacitance of 109.4 mF cm^−2^. This work demonstrates an effective strategy for enhancing the alkaline HER performance of Fe-based phosphides through the combination of Co incorporation and dealloying-derived nanoporous architecture.

## 1. Introduction

With the growing demand for sustainable energy and the increasing concerns over environmental pollution caused by fossil fuel consumption, hydrogen has been widely regarded as a promising clean energy carrier because of its high energy density and carbon-free utilization. Among various hydrogen production technologies, electrochemical water splitting is considered one of the most attractive routes [1,2,3,4]. However, the overall efficiency of water electrolysis critically depends on highly active and durable electrocatalysts. Although noble-metal-based catalysts, such as Pt, exhibit excellent HER activity, their high cost and limited availability severely hinder large-scale application. Therefore, earth-abundant transition-metal-based electrocatalysts have attracted extensive attention as low-cost and sustainable alternatives to noble-metal catalysts for water splitting [5,6,7,8,9].

Transition-metal phosphides (TMPs) have emerged as promising HER electrocatalysts because of their structural diversity, tunable electronic properties, and favorable metal–phosphorus interactions [10,11,12]. Previous studies have demonstrated that TMPs, such as Ni_2_P, CoP, FeP, and MoP, can exhibit considerable HER activity, indicating their promise as noble-metal-free catalysts [13,14,15,16,17]. Nevertheless, single metal phosphides still suffer from several intrinsic limitations, including insufficient active sites, sluggish interfacial charge transfer kinetics, and unfavorable product desorption, all of which restrict their further catalytic improvement [18,19,20].

To further enhance the HER performance of single metal phosphides, two general strategies are widely considered effective: improving the intrinsic activity of active sites and increasing the number of accessible active sites [21,22]. The incorporation of a second additive is a promising approach for enhancing the intrinsic activity of active sites by modulating the local electronic environment of metal phosphides [23,24,25]. Nonmetal elements, such as B, C, N, and F, can be incorporated into metal phosphides, and their relatively high electronegativity can increase the valence state of metal atoms and thereby regulate the local electronic structure of the metal centers. For example, Wang et al. [26] introduced N into CoP_2_ and obtained significantly enhanced alkaline HER activity, requiring only 64 mV to achieve 10 mA cm^−2^ in 1.0 M KOH. Theoretical calculations indicated that N substitution for P facilitated electron transfer and optimized the adsorption free energies of H*, H_2_O, and OH*, thereby promoting the HER process. Similarly, Gao et al. [27] demonstrated that surface-confined B doping in CoP could regulate the valence state of Co centers and improve alkaline HER kinetics. In addition to nonmetal dopants, second metal elements can also serve as effective additives. For example, Wang et al. [28] introduced Cu into CoP nanoparticles, where Cu doping not only intensified the Kirkendall effect to form double-shell hollow structures but also optimized OH* adsorption at the Cu-CoOH/Cu-CoP interface, thereby improving the intrinsic OER activity of CoP-based phosphides. Liu et al. [29] introduced Ni and Mn into FeP nanoarrays to manipulate the d-band centers of transition-metal phosphides, which optimized H* adsorption and promoted *O-to-*OOH conversion, leading to enhanced HER/OER activity and robust overall water-splitting performance.

Another effective route for improving metal phosphides is to increase the number of accessible active sites. Compared with many other preparation methods, dealloying is considered an efficient strategy because of its simplicity and controllability [30,31,32]. For example, Xu et al. [33] prepared carbon-doped nanoporous Co_2_P through electrochemical dealloying, in which the porous framework provided abundant accessible active sites and improved mass transport. Xia et al. [34] used dealloying to fabricate self-supported Mo-doped hierarchical porous Ni-P catalysts, in which the multistage porous framework provided abundant accessible active sites and promoted charge/mass transport.

Owing to the earth abundance and low cost of iron, Fe-based phosphides are considered promising HER catalysts for practical applications. Fe-P compounds have exhibited appreciable catalytic activity over a wide pH range, highlighting their potential for large-scale hydrogen production [35,36,37]. In particular, Fe_3_P has been reported to show more favorable HER behavior than FeP and Fe_2_P among iron phosphides [38]. However, monometallic Fe_3_P still suffers from limited electronic modulation and insufficient exposure of accessible active sites, which restrict its alkaline HER performance. In this work, Co-doped nanoporous Fe_3_P was constructed through precursor-alloy engineering followed by electrochemical dealloying. Specifically, Fe_75_Co_5_P_20_ precursor alloys were first prepared to form a dual-phase structure containing a Co-incorporated Fe_3_P phase and an Fe-rich phase. During the subsequent electrochemical dealloying process, the Fe-rich phase was selectively dissolved, while the Co-doped Fe_3_P framework was retained, leading to a continuous nanoporous architecture rather than merely surface pits. Nanoporous Fe_3_P prepared from Fe_80_P_20_ was used as the reference to clarify the role of Co incorporation. Through systematic structural characterization and electrochemical measurements, the relationship among Co incorporation, phase-selective dealloying, nanoporous-framework formation, and alkaline HER performance was investigated. The results show that Co incorporation, combined with the dealloying-derived nanoporous framework, provides a feasible strategy for improving the HER activity of Fe-based phosphides.

## 2. Materials and Methods

### 2.1. Preparation of Alloys

The precursor alloys were prepared by high-frequency induction melting and melt-spinning methods. Bulk Fe, Fe_2_P powders, and bulk Co for the Fe_75_Co_5_P_20_ alloy and bulk Fe, Fe_2_P powders for Fe_80_P_20_ were weighed according to the designed atomic ratio and melted in an induction furnace under a high-purity Ar atmosphere to obtain a homogeneous alloy ingot. The alloy ingots were subsequently remelted in quartz tubes and then rapidly quenched onto a rotating copper wheel under a pressure difference of 0.03 MPa, with the wheel speed fixed at 3000 rad min^−1^, to obtain the corresponding precursor ribbons. Then, nanoporous Co-Fe_3_P and Fe_3_P were obtained from the as-spun ribbons by electrochemical dealloying in 1 M HCl at an applied potential of −0.1 V vs. Ag/AgCl for 1 h. After dealloying, the samples were thoroughly washed with pure water and ethanol at least three times, and then collected in the vacuum oven for subsequent structural characterization and electrochemical testing.

### 2.2. Characterization

The surface morphology and microstructural features were verified using scanning electron microscopy (SEM, Hitachi S-4800, Tokyo, Japan). The crystal structures and phase compositions of the samples were analyzed by X-ray diffraction (XRD, Bruker D8 Advance, Karlsruhe, Germany) using Cu Kα radiation (λ = 0.15418 nm) with a step size of 0.02° and a scanning rate of 2.4° min^−1^. The surface elemental composition and chemical states of the samples were analyzed by X-ray photoelectron spectroscopy (XPS, Axis Supra, Kratos Analytical, Manchester, UK). Details of nanostructures were investigated using a transmission electron microscope (TEM, JEOL JEM-2100 M, Tokyo, Japan).

### 2.3. Electrochemical Measurements

The electrochemical measurements were performed in an electrochemical workstation using a standard three-electrode cell. The as-prepared self-supported np-Co-Fe_3_P and np-Fe_3_P ribbons were directly used as the working electrodes, with a platinum mesh and a saturated Ag/AgCl electrode serving as the counter and reference electrodes, respectively. The exposed geometric area of each working electrode was 0.2 cm^2^, and the current density was normalized to this area. All measurements were performed in 1 M KOH (pH = 14) at room temperature. The HER polarization curves were obtained by linear sweep voltammetry (LSV) at a scan rate of 5 mV s^−1^ with 80% iR compensation. Prior to the LSV measurements, several cyclic voltammetry (CV) scans were conducted until a stable electrochemical response was obtained. To reduce experimental error, each electrochemical measurement was repeated at least three times under identical conditions. The measured potentials versus Ag/AgCl were converted to the reversible hydrogen electrode (RHE) according to the Nernst equation:

(1) $$E_{\mathrm{RHE}}=E_{\mathrm{Ag}/\mathrm{AgCl}}+0.197+0.059\times \mathrm{pH}$$

Electrochemical impedance spectroscopy (EIS) was conducted at a cathodic overpotential of −0.2 V vs. RHE over a frequency range of 100 kHz to 0.1 Hz with an AC amplitude of 5 mV. The EIS results were used to evaluate the interfacial charge-transfer behavior during HER. In general, a smaller semicircle diameter in the Nyquist plot indicates a lower charge-transfer resistance (R_ct_).

The electrochemical double-layer capacitance (C_dl_) was determined from CV curves recorded in a non-faradaic potential region of 0.125–0.225 V vs. RHE at scan rates of 20–100 mV s^−1^. The Tafel slope was obtained by fitting the linear region of the polarization curves according to the Tafel equation:

(2) $$\eta =a+b\log\left|j\right|$$

where η is the overpotential, b is the Tafel slope, and j is the current density.

The long-term HER stability was evaluated by the chronopotentiometry at a constant current density of 100 mA cm^−2^ for 20 h in 1 M KOH.

## 3. Results and Discussion

### 3.1. Structure Characterization

**Figure 1:**
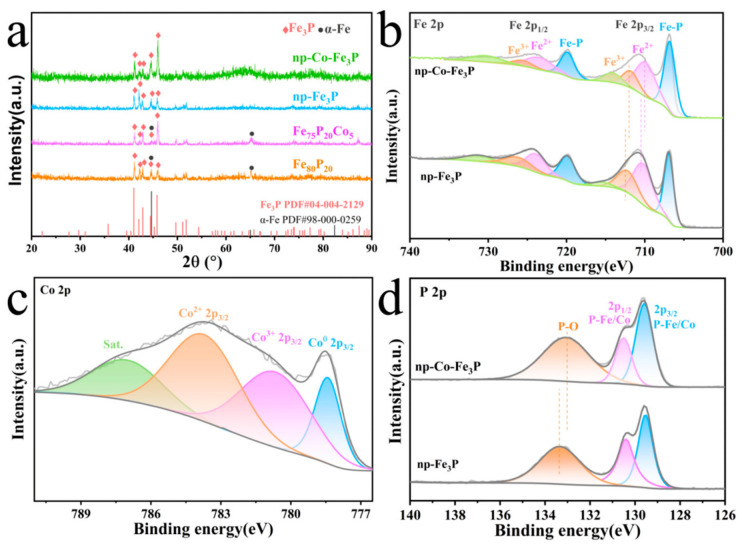
(**a**) XRD patterns of np-Co-Fe_3_P and np-Fe_3_P before and after dealloying; XPS spectra of (**b**) Fe 2p, (**c**) Co 2p, and (**d**) P 2p.

Figure 1a shows the XRD patterns of the alloy ribbons before and after dealloying. Before dealloying, both the Fe_75_Co_5_P_20_ and Fe_80_P_20_ precursor ribbons are mainly composed of Fe_3_P and α-Fe, which accords with the Fe-P phase diagram [39]. The dense group of reflections in the 40–46° range, located at 41.38°, 42.26°, 42.90°, 44.68°, and 46.02°, can be indexed to the (321), (330), (112), (420), and (141) planes of Fe_3_P, respectively. In addition, the peaks at 44.68° and 65.22° are assigned to the (110) and (200) planes of α-Fe, respectively. After electrochemical dealloying, the XRD patterns of np-Co-Fe_3_P and np-Fe_3_P exhibit only the characteristic reflections of Fe_3_P, while no obvious diffraction peaks of α-Fe were detected. This result indicates that the α-Fe phase was selectively removed during the dealloying process, whereas the Fe_3_P phase was retained in the nanoporous framework.

The surface chemical compositions and valence states of the dealloyed catalysts are further examined by XPS. In the Fe 2p spectrum of np-Co-Fe_3_P (Figure 1b), the peaks at 707.0 and 720.0 eV are assigned to Fe-P species, whereas the peaks at 710.3 and 724.2 eV correspond to Fe^2+^2p3/2 and 2p1/2, respectively, and those at 713.2 and 728.9 eV are attributed to Fe^3+^2p3/2 and 2p1/2. These results indicate the coexistence of Fe-P species and oxidized Fe species, suggesting that the phosphide framework is retained while the surface is partially oxidized. Compared with np-Fe_3_P, the oxidized Fe peaks of np-Co-Fe_3_P shift slightly toward lower binding energy, suggesting that Co incorporation may modify the local coordination and electronic environment around Fe [11]. The P 2p spectrum (Figure 1d) shows two peaks at 129.5 and 130.5 eV, which are assigned to P 2p3/2 and P 2p1/2 of metal phosphides, respectively. In addition, the peak near 133 eV is attributed to P-O species formed by surface oxidation [40]. Compared with np-Fe_3_P, the P-O component of np-Co-Fe_3_P shifts slightly toward lower binding energy, indicating that Co incorporation modifies the local electronic environment around P and may increase the electron density of surface oxidized P species. The Co 2p3/2 core spectrum of np-Co-Fe_3_P (Figure 1c) can be deconvoluted into three main peaks at 778.4, 780.8, and 783.9 eV, which are assigned to Co^0^, Co^3+^, and Co^2+^, respectively, together with a satellite peak at 787.2 eV. This result further confirms the successful incorporation of Co into the Fe_3_P framework [41,42].

**Figure 2:**
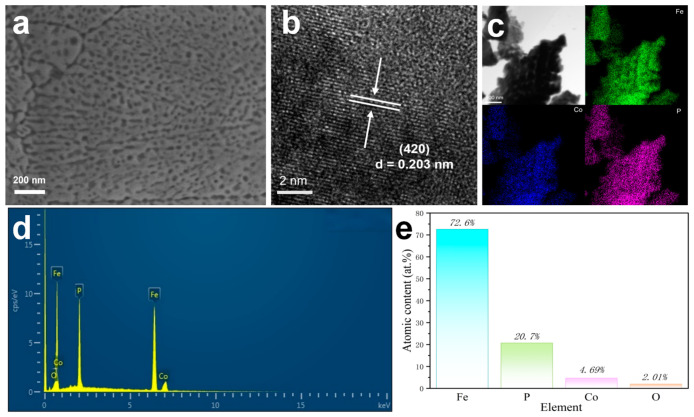
(**a**) SEM image, (**b**) HRTEM image, (**c**) elemental mapping images, (**d**) EDX spectrum, and (**e**) atomic contents of np-Co-Fe_3_P.

The SEM image (Figure 2a) demonstrates that the np-Co-Fe_3_P consists of numerous interconnected nanoscale channels, forming a three-dimensional nanoporous structure. Moreover, the hole size estimated from the SEM image is mainly distributed in the range of 10–26 nm, with an average size of approximately 19.4 ± 5.5 nm. Such a nanoporous framework is expected to provide a larger electrochemically accessible surface and more exposed active sites, which likely contribute to the improved electrochemical performance. The high-resolution TEM image (Figure 2b) reveals well-defined lattice fringes with an interplanar spacing of approximately 0.203 nm, which is assigned to the (420) plane of Fe_3_P. In addition, the corresponding mapping images (Figure 2c) show that Fe, Co, and P are homogeneously distributed throughout the np-Co-Fe_3_P sample. The EDX spectrum (Figure 2d), together with the corresponding atomic content analysis (Figure 2e), further confirms the successful incorporation of Co into the nanoporous Fe_3_P-based framework.

### 3.2. Hydrogen Evolution Performance of the Catalysts

**Figure 3:**
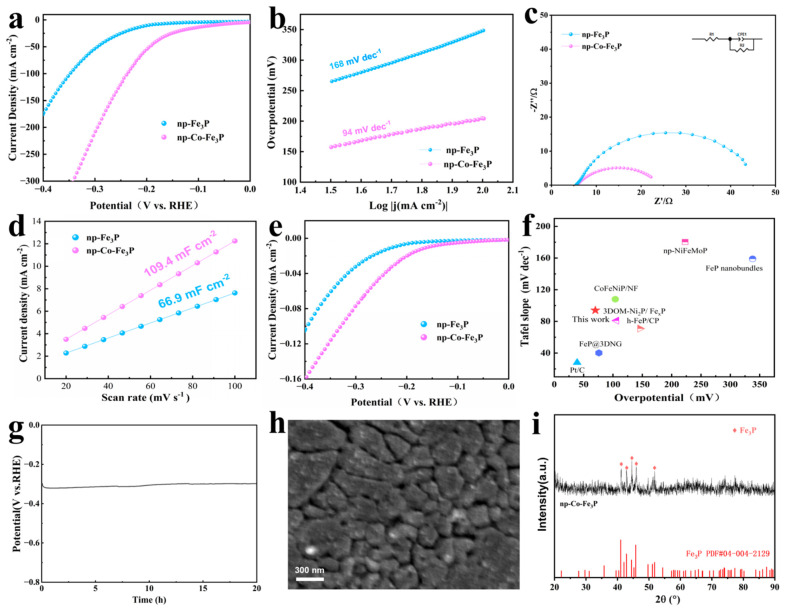
Electrochemical HER performance of prepared catalysts in 1 M KOH. (**a**) LSV curves of HER. (**b**) Tafel plots. (**c**) Nyquist plots. (**d**) Capacitive current density extracted at 0.17 V vs. RHE plotted against scan rate. (**e**) ECSA-normalized LSV curves of HER. (**f**) Comparison of overpotential and Tafel slopes of np-Co-Fe_3_P with other reported HER electrocatalysts. (**g**) Chronopotentiometric curve of np-Co-Fe_3_P at 100 mA cm^−2^. (**h**) SEM image of np-Co-Fe_3_P after the stability test. (**i**) XRD image of np-Co-Fe_3_P after the stability test.

To evaluate the HER performance of the prepared catalysts, electrochemical measurements were carried out in 1.0 M KOH using a three-electrode system, in which the as-prepared samples were directly used as self-supported working electrodes. As shown in Figure 3a, Co incorporation markedly improves the HER activity. Specifically, np-Co-Fe_3_P requires an overpotential of only 70 mV to reach 10 mA cm^−2^, which is much lower than that of np-Fe_3_P (199 mV), indicating substantially enhanced catalytic activity after Co doping. The HER kinetics are further evaluated from the corresponding Tafel plots (Figure 3b). The Tafel slope of np-Co-Fe_3_P is 94 mV dec^−1^, which is significantly lower than that of np-Fe_3_P (168 mV dec^−1^), confirming the accelerated HER kinetics of the Co-containing catalyst. Electrochemical impedance spectroscopy further supports this trend. As shown in Figure 3c, np-Co-Fe_3_P exhibits a much smaller semicircle than np-Fe_3_P, with fitted Rct values of 17.9 Ω and 40.4 Ω for np-Co-Fe_3_P and np-Fe_3_P, respectively. This indicates that np-Co-Fe_3_P has a lower charge-transfer resistance and faster interfacial electron-transfer kinetics during HER. In addition, the double-layer capacitance values derived from the non-faradaic CV curves (Figure 3d) are 109.4 mF cm^−2^ for np-Co-Fe_3_P and 66.9 mF cm^−2^ for np-Fe_3_P, suggesting that Co incorporation leads to a larger electrochemically active surface area and more accessible active sites. Furthermore, the ECSA normalized LSV curves (Figure 3e) show that np-Co-Fe_3_P still maintains higher normalized HER activity, indicating that Co incorporation also contributes to improved intrinsic catalytic activity. The durability of np-Co-Fe_3_P was examined by chronopotentiometry at 100 mA cm^−2^ for 20 h, as shown in Figure 3g. The np-Co-Fe_3_P catalyst reveals negligible attenuation and favorable stability under high-current operation. Moreover, the post-test SEM image (Figure 3h) shows that the porous framework is still retained after long-term HER testing, although slight surface coarsening can be observed. The post-stability XRD pattern (Figure 3i) still displays the characteristic diffraction peaks of Fe_3_P, and no obvious new crystalline phases are detected after the HER stability test. This result indicates that np-Co-Fe_3_P maintains reasonable structural integrity during prolonged electrolysis. Overall, the improved HER performance of np-Co-Fe_3_P is closely associated with Co incorporation, which is accompanied by lower overpotential, faster charge-transfer kinetics, and a larger C_dl_.

## 4. Conclusions

In this work, a Co-doped nanoporous Fe_3_P self-supported electrode was successfully fabricated by melt spinning followed by electrochemical dealloying of Fe_75_Co_5_P_20_ precursor ribbons. The dealloying process removed the α-Fe phase and preserved the Fe_3_P framework, leading to a porous structure with abundant accessible surface sites. Compared with np-Fe_3_P, np-Co-Fe_3_P exhibited significantly improved HER performance in 1.0 M KOH, requiring an overpotential of only 70 mV at 10 mA cm^−2^, together with a smaller Tafel slope of 94 mV dec^−1^, lower charge-transfer resistance, and a larger double-layer capacitance of 109.4 mF cm^−2^. The improved performance demonstrates that Co incorporation is beneficial for enhancing the HER behavior of nanoporous Fe_3_P by improving reaction kinetics and increasing the electrochemically active surface area. This work provides a feasible strategy for developing efficient Fe-based phosphide electrocatalysts for alkaline hydrogen evolution.

## Data Availability

The original contributions presented in this study are included in the article. Further inquiries can be directed to the corresponding author.
